# Disclosing conflicts of interest in patient decision aids

**DOI:** 10.1186/1472-6947-13-S2-S3

**Published:** 2013-11-29

**Authors:** Michael J Barry, Evelyn Chan, Benjamin Moulton, Sunita Sah, Magenta B Simmons, Clarence Braddock

**Affiliations:** 1 Informed Medical Decisions Foundation, 40 Court Street, Suite 300, Boston, Massachusetts, 02108, USA; 2 Department of Medicine, University of Texas Health Sciences Center at Houston, 6431 Fannin Street, Houston, Texas, 77030, USA; 3 Informed Medical Decisions Foundation, 40 Court Street, Suite 300, Boston, Massachusetts, 02108, USA; 4 Department of Strategy, Economics, Ethics, and Public Policy, Georgetown University, 37th and O Street, Rafik B. Hariri Building, Washington, District of Columbia, 20057, USA; 5 Edmond J. Safra Center for Ethics, Harvard University, 124 Mount Auburn Street, Suite 520N, Boston, Massachusetts, 02138, USA; 6 Orygen Youth Health Research Centre, Centre for Youth Mental Health, The University of Melbourne, 35 Poplar Road, Parkville Victoria 3052, Australia; 7 Stanford Center for Medical Education Research, Stanford School of Medicine, 251 Campus Drive MSOB X333, 5404, Stanford, CA 94305-5404, USA

## Abstract

**Background:**

In 2005, the International Patient Decisions Aid Standards (IPDAS) Collaboration developed quality criteria for patient decisions aids; one of the quality dimensions dealt with disclosure of conflicts of interest (COIs). The purposes of this paper are to review newer evidence on dealing with COI in the development of patient decision aids and to readdress the theoretical justification and definition for this quality dimension.

**Methods:**

The committee conducted a primary systematic literature review to seek published research addressing the question, "What is the evidence that disclosure of COIs in patient decision aids reduces biased decision making?" A secondary literature review included a systematic search for recent meta-analyses addressing COIs in other spheres of health care, including research and publication, medical education, and clinical care.

**Results:**

No direct evidence was found addressing this quality dimension in the primary literature review. The secondary review yielded a comprehensive Institute of Medicine report, as well as four relevant meta-analyses addressing disclosure of COIs in health care. They revealed a broad consensus that disclosure of COIs is desirable in such areas as research publication, guideline development, medical education, and clinical care.

**Conclusions:**

The committee recommends the criteria that are currently used to operationally define the quality dimension “disclosing conflicts of interest” be changed as follows (changes in italics):

Does the patient decision aid:

• report *prominently and in plain language* the source of funding to develop or exclusively distribute the patient decision aid?

• report *prominently and in plain language* whether funders, authors, or their affiliations, stand to gain or lose by choices patients make after using the patient decision aid?

Furthermore, based on a consensus that simple disclosure is insufficient to protect users from potentially biased information, the committee recommends that the IPDAS Collaboration consider adding the following criterion when the IPDAS consensus process is next conducted: “Does the patient decision aid:

• report that no funding to develop or exclusively distribute the patient decision aid has been received from commercial, for-profit entities that sell tests or treatments included as options in the patient decision aid?”

## Background

Patient decision aids have been defined as tools designed to help people participate in decision making about health care options. They provide information on the options and help patients clarify and communicate the personal value they associate with different features of the options [1]. In 2005, the International Patient Decisions Aid Standards (IPDAS) Collaboration developed quality criteria for patient decisions aids using a two stage web-based Delphi process [2]. The process involved 122 representatives of four stakeholder groups (researchers, practitioners, patients, and policy makers) from 14 countries, and was informed by evidence reviews on each potential quality dimension. One of the quality dimensions addressed by this process dealt with disclosure of conflicts of interest (COIs) in the development and distribution of patient decision aids.

For the second round of voting in the IPDAS Collaboration’s Delphi process, the conceptual definition of “disclosing conflicts of interest” was for patient decision aids to be open and honest in stating:

• the funding source for creating and producing patient decision aids,

• the financial support for practitioners who are responsible for creating the patient decision aid, and

• the affiliations of patient decision aid developers that might influence the content of patient decision aids.

In the IPDAS Collaboration’s checklist that eventually emerged out of the Delphi process, two operational criteria for the disclosing COIs dimension emerged:

Does the patient decision aid:

• report source of funding to develop and distribute the patient decision aid?

• report whether authors or their affiliations stand to gain or lose by choices patients make after using the patient decision aid?

The purposes of this paper are to review newer evidence on dealing with COIs in the development of patient decision aids that has emerged since 2005, to readdress the theoretical justification and definition for this quality dimension, and to discuss emerging issues that might cause the original consensus criteria to be rethought.

## Theoretical rationale for evaluating patient decision aids on this quality standard

A definition of “conflict of interest” that is commonly used by medical journals is, “a set of conditions in which professional judgment concerning a primary interest (such as patients’ welfare or the validity of research) tends to be unduly influenced by a secondary interest (such as financial gain)” [3]. Practitioners and scientists who read research reports and reviews in medical journals represent a relatively well-educated audience, yet journal editors remain concerned about adequate disclosure of potential COIs by the authors of those papers [4,5]. Users of patient decision aids often come from the lay public, and their ability to detect and evaluate the influence of potential COIs on the content of the programs they use may be poorer compared with medical professionals. Thus, patients may be more vulnerable to any bias incorporated into patient decision aids than medical professionals are to biases that may enter into scientific papers in medical journals. Moreover, patient decision aids may be developed and disseminated for use by patients or members of the public without the benefit of scrutiny by independent peer reviewers or an independent editor, as would generally be the case for papers in most peer-reviewed medical journals.

Therefore, it seems reasonable that requirements for disclosure of potential COIs should be at least as stringent as disclosure requirements for medical journals. In addition, for a lay audience, explaining how the financial interests of any commercial funders relate to the patient decision aid’s content seems like a reasonable approach to help patients and practitioners decide whether a program is likely to be biased by such interests. While a professional viewer might know that a particular funder makes or sells a product described as an option in the patient decision aid, a lay viewer might not.

## Empirical evidence

### Primary literature review

The committee conducted a systematic literature review to seek published research addressing the question, "What is the evidence that disclosure of COIs in patient decision aids reduces biased decision-making?" The PubMed database was searched back ten years from the date of the search, May 11, 2011. Several preliminary searches were done to inform the development of a search strategy maximizing sensitivity at the cost of some specificity. The final search strategy used the terms: ((shared decision making) OR (patient centered decision) OR (decision aid) OR (decision support) AND (disclos* OR (conflict of interest)).

This search yielded 874 titles. The titles were scanned by at least two committee members, and abstracts or full text articles as necessary were retrieved for articles flagged as potentially relevant to the research question by either reviewer. Ultimately, two articles were judged to be relevant. One (reassuringly) was the original IPDAS paper in the *British Medical Journal* describing the development and validation of the IPDAS quality criteria, including disclosure of COIs [2]. The other paper, judged to be only marginally relevant, was a systematic review of decision support technologies for the decision about amniocentesis [6]. While this paper documented that five patient decision aids on this topic generally included disclosure of potential COIs, there was no information on the effect of those disclosures on the patients’ decisions.

### Secondary literature review

Based on this primary literature review, the committee concluded that, as in the IPDAS Collaboration’s original overview of the related empirical evidence, there was no direct evidence to support this quality criterion. The rationale would need to continue to be based on indirect evidence on the importance of disclosure of COIs in other spheres of health care, including research and publication, medical education, and clinical care. A complete systematic review of all this evidence was beyond the scope of this project, but the committee members’ personal bibliographies were reviewed for summaries of this evidence, and a search was conducted on September 29, 2011 going back 5 years for systematic reviews addressing COIs in medical research and clinical care. Using the search terms (disclos* OR (conflict of interest)) AND systematic review, we identified 1305 titles of which 31 were selected as potentially relevant. Review of abstracts or full text articles yielded four relevant systematic reviews. The key new evidence uncovered by the systematic review and the members’ bibliographies is summarized below.

#### Reports

A comprehensive report on COIs by the Institute of Medicine (IOM) of the National Academies in the United States was identified, which was judged to be a key new reference supporting the original IPDAS criteria on COI [7]. In 2009, the IOM of the National Academies in the United States released a comprehensive report on COIs in medical research, education, and practice. The committee summarized the problem as follows: “…financial ties between medicine and industry may create COIs. Such conflicts present the risk of undue influence on professional judgments and thereby may jeopardize the integrity of scientific investigations, the objectivity of medical education, the quality of patient care, and the public’s trust in medicine.”

The key recommendations of the report included the following: “The committee recommends that medical institutions—including academic medical centers, professional societies, patient advocacy groups, and medical journals—establish conflict of interest policies that require disclosure and management of both individual and institutional financial ties to industry.”

The report went on to acknowledge that disclosure was only the first step in identifying and responding to COIs. The committee recommended a national reporting program accessible to the public disclosing industry payments to physicians, researchers, health care institutions, professional societies, patient advocacy groups, and medical education providers that would permit verification of adequate disclosure by patients and the public.

In addition, the report singled out concerns about COIs in the development of clinical practice guidelines, stating: “Clinical practice guidelines influence physician practice, quality measures, and insurance coverage decisions… The committee recommends that professional societies and other groups that develop practice guidelines not accept direct industry funding for guideline development and generally exclude individuals with COIs from panels that draft the guidelines. In addition, these groups should make public their COI policies, their funding sources, and any financial relationships panel members have with industry.” This comment is particularly important, as patient decision aids can be viewed as practice guideline information for consumption by patients facing health decisions.

#### Editorials

In his 2006 editorial, Richard Smith pointed out how ubiquitous COIs were in health care [4]. He opined about the evidence that COIs affect the referral of patients and the interpretations of studies, as well as the importance of disclosure in health care and the threshold levels of conflict that might rule out people from referring patients or writing editorials. In a 2008 editorial, editors of the Journal of the American Medical Association wrote, “*Primum non nocere* does not only hold true for physicians directly treating patients, but also holds true for all involved in medical research, biomedical publication, and medical education [5].”

In fact, by 2008, most medical journals with high impact factors had author COI policies, although how they were operationalized varied considerably [8]. Similarly, a survey of US medical schools in 2007 revealed that most had COI policies addressing clinical care, though they were rather weak [9].

#### Empirical evidence from systematic reviews

Despite the strong opinions expressed in the IOM report and these editorials, there is relatively limited empirical evidence on the effect of COI policies on mitigating negative effects of conflicts of interest on behavior. As the IOM report summarizes, “Empirical data on conflict of interest policies are limited, have methodological shortcomings, and tend to focus on academic institutions.” For example, several studies have shown conflicting results in terms of physicians’ financial disclosures on patient trust [10,11], demonstrating that how the disclosure is given and interpreted is critical. A recent randomized trial showed that disclosure of research funding significantly influenced how physicians viewed the methodological quality of the trial and their willingness to change their practice based on the results [12].

Our search for systematic reviews yielded evidence on some of these aspects of disclosure. The strongest empirical evidence on the effect of COIs in medicine comes from the domain of publication of research findings. A systematic review that identified 57 drug trials published between 2002 and 2009 found that trials funded by manufacturers yielded more favorable results than in independently financed trials; this difference was not explained by differences in methodological quality between the two groups of trials [13]. The results of this review were consistent with earlier systematic reviews on this topic; 17 systematic reviews published between 2003 and 2006 (either addressing drug trials in general or trials of specific drug classes, such as antidepressants) found an association between industry support and published pro-industry trials, while two did not [14]. However, studies of the effect of disclosing funding sources for published research on perceived credibility of the research among practicing clinicians have yielded mixed results [15,16].

Another area with empirical evidence supporting the impact of medical COIs on behavior is the effect of information from pharmaceutical companies on clinicians’ prescribing behavior. In a systematic review of studies of this influence published up to 2008, 38 studies reported an association between exposure to information from pharmaceutical companies and lower-quality prescribing behavior, while 13 did not [17].

Furthermore, Licurse and colleagues conducted a systematic review of studies of patients’, research participants’, and journal readers’ attitudes toward financial disclosure [18]. A search of studies from 1988-2009 found only 20 relevant articles. Six addressed patients’ reactions to disclosure of financial ties in clinical care, and five addressed readers’ reactions to disclosure of financial ties in research. Ten addressed the importance of disclosing financial ties in clinical care and research. In clinical care, patients believed financial ties decreased the quality and increased the cost of clinical care, and in research, financial ties affected perceptions of study quality. Most studies found that the majority of patients and research participants believed financial ties should be disclosed.

In addition to these systematic reviews, a recent narrative review cited evidence that financial relationships with referral sources, health insurers, and the drug and device industries can bias physician decision making [19].

## Discussion

In summary, based on the IOM report and the data from the systematic reviews on this topic, there is a broad consensus in medicine that disclosure of COIs is desirable in such areas as research publication, guideline development, medical education, and clinical care. Moreover, people—whether as patients or research subjects—generally feel financial ties between clinicians or researchers and industry should be disclosed. Patient decision aids are designed to provide patients with unbiased information about their options and the pros and cons of those options to help them work with their clinicians to make informed, value-based decisions for their personal health care. The potential for bias in patient decision aids due to COIs seems as great as in these other areas of health care. Moreover, the phenomenon of patients making suboptimal decisions based on biased information might have even more drastic consequences, particularly for “high stakes” decisions about diagnosis and treatment of serious medical conditions. Therefore, despite the lack of direct evidence pertaining to patient decision aids, the committee strongly felt that it remains important to require disclosure of potential COIs as a quality criterion, both in terms of funding for the development of patient decision aids, as well as funding for the authors responsible for the content of the aids. To make these disclosures useful, however, the committee judged that the operational definitions of this quality dimension—as reflected in the two current assessment criteria—be modified slightly. The committee judged that these criteria should not be considered met unless users could readily find the disclosure information and be likely to interpret it correctly. Therefore, the committee recommended that disclosure information in patient decision aids be provided *prominently and in plain language*.

By “prominently”, the committee means that disclosure information should be provided along with the body of clinical information in the decision aid and not separately—say, on a separate web site or in a separate technical document. Disclosure information should be presented in a similar font to clinical information and not in the “fine print,” whether the decision aid is provided in paper, video, or Internet-based format.

By “plain language”, the committee means that the disclosure information should be provided in simple, straightforward language and not in technical jargon that would be difficult for lay users to comprehend.

The committee has also clarified the language around the declaration of sources of funding for decision aid distribution. Once produced, a patient decision aid may be sold or licensed for use to many different entities, and declaring all such relationships would be impractical. However, when a patient decision aid is produced for exclusive distribution by one funder, even if that funder does not pay for the development, that funding should be disclosed.

Going further, committee members were uncomfortable, even in the absence of direct evidence addressing this quality dimension, that simple disclosure is sufficient to protect lay viewers from potentially biased information in patient decision aids. Lay viewers may not appreciate, to the same degree that health care professionals might, the potential for bias when commercial, for-profit entities provide funding for patient decision aids that discuss their own products. This discomfort on the part of the committee parallels a growing sense in the literature [20], particularly as reflected in the recent comprehensive IOM report [7], that disclosure is necessary but not sufficient to avoid bias in research publication, guideline development, medical education, and clinical care.

Based on the interim review of the evidence, the committee decided to recommend to the IPDAS Collaboration that the assessment criteria for this quality dimension be extended with the addition of a third criterion. The committee felt that disclosure alone is not sufficient to protect lay users from potential bias resulting from funding of patient decision aids by commercial, for-profit entities that produce or distribute tests or treatments included as options. This third quality criterion would not be awarded unless the disclosure information provided revealed that the patient decision aid’s developers had received no funding from commercial, for-profit entities that produce or distribute tests or treatments included as options in the patient decision aid.

Further research is clearly necessary, especially research applying directly to the relationship between funding sources and biases in patient decision aids. This work will need to include fundamental methodological studies of how bias in patient decision aids can be accurately and reliably judged. In addition, more research is needed on the necessary level of detail and prominence of disclosure statements in patient decision aids, particularly in multimedia presentations, as well as how viewers interpret them. The IPDAS Collaboration’s promotion of routine disclosure of potential COIs in patient decision aid development can help facilitate this research.

## Conclusion

Based on a review of the evidence, the committee recommends the criteria which represent the operational definition of the disclosure of conflict of interest quality dimension be changed as follows (change in italics): Does the patient decision aid:

• report *prominently and in plain language* the source of funding to develop or exclusively distribute the patient decision aid?

• report *prominently and in plain language* whether funders, authors or their affiliations stand to gain or lose by choices patients make after using the patient decision aid?

Furthermore, based on a consensus that simple disclosure is insufficient to protect users from potentially biased information, the committee recommends that the IPDAS Steering Committee consider adding the following criterion when the IPDAS consensus process is next conducted: “Does the patient decision aid:

• report that no funding to develop or exclusively distribute the patient decision aid has been received from commercial, for-profit entities that sell tests or treatments included as options in the patient decision aid?”

## List of abbreviations used

COI: Conflict of interest; IOM: Institute of Medicine; IPDAS: International Patient Decision Aid Standards

## Competing interests

Michael Barry and Benjamin Moulton receive salary support as president and senior legal advisor, respectively, of the nonprofit Informed Medical Decisions Foundation. Clarence Braddock receives honoraria as a board member of the Informed Medical Decisions Foundation. The Foundation receives royalty and project revenue from a for-profit company, Health Dialog, with which it coproduces patient decisions aids that are in turn licensed to users. All other authors declare no competing interests.

## Authors' contributions

All authors contributed to the design of the search strategy and review of the results of the searches. Dr. MJB drafted the manuscript and all authors contributed to critical revisions for important intellectual content that led to the final version. All authors read and approved the final version as submitted.

## Declarations

Publication of this supplement was funded by an unrestricted grant from the Informed Medical Decisions Foundation and by the John D. Stoeckle Center for Primary Care Innovation at Massachusetts General Hospital. Administrative and editorial support for this supplement was also provided by The University of Texas MD Anderson Cancer Center.

This article has been published as part of *BMC Medical Informatics and Decision Making* Volume 13 Supplement 2, 2013: The International Patient Decision Aid Standards (IPDAS) Collaboration’s Quality Dimensions: Theoretical Rationales, Current Evidence, and Emerging Issues. The full contents of the supplement are available online at http://www.biomedcentral.com/bmcmedinformdecismak/supplements/13/S2.
